# Supplementary figures and images for: Dynamic Effective Connectivity of Inter-Areal Brain Circuits
Source: PLoS Comput Biol. 2012 Mar 22;8(3):e1002438. doi: 10.1371/journal.pcbi.1002438 (PMC3310731; doi:10.1371/journal.pcbi.1002438)

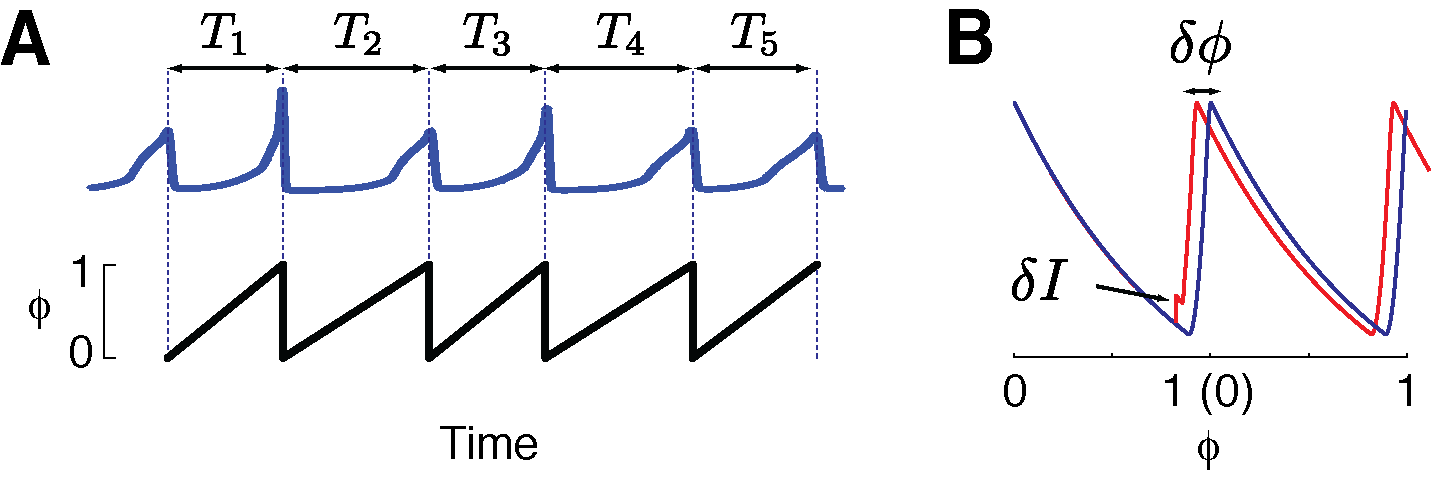

Supplement: Figure S1 — Phase reduction and phase response. A: oscillating time-series (in the example, a “LFP” time-series from the network model) can be described in terms of phase, even if they are not periodic in strict sense, by interpolating linearly an instantaneous empiric phase variable to the oscillation cycles (generally of unequal lengths). B: the application of a pulse current induces a shift in the oscillation phase of the ongoing oscillation (in the example, a rate trace from the rate model). The amplitude of the induced shift depends on the phase of the ongoing oscillation at which the perturbation is applied. (TIFF) [file pcbi.1002438.s001.tiff]

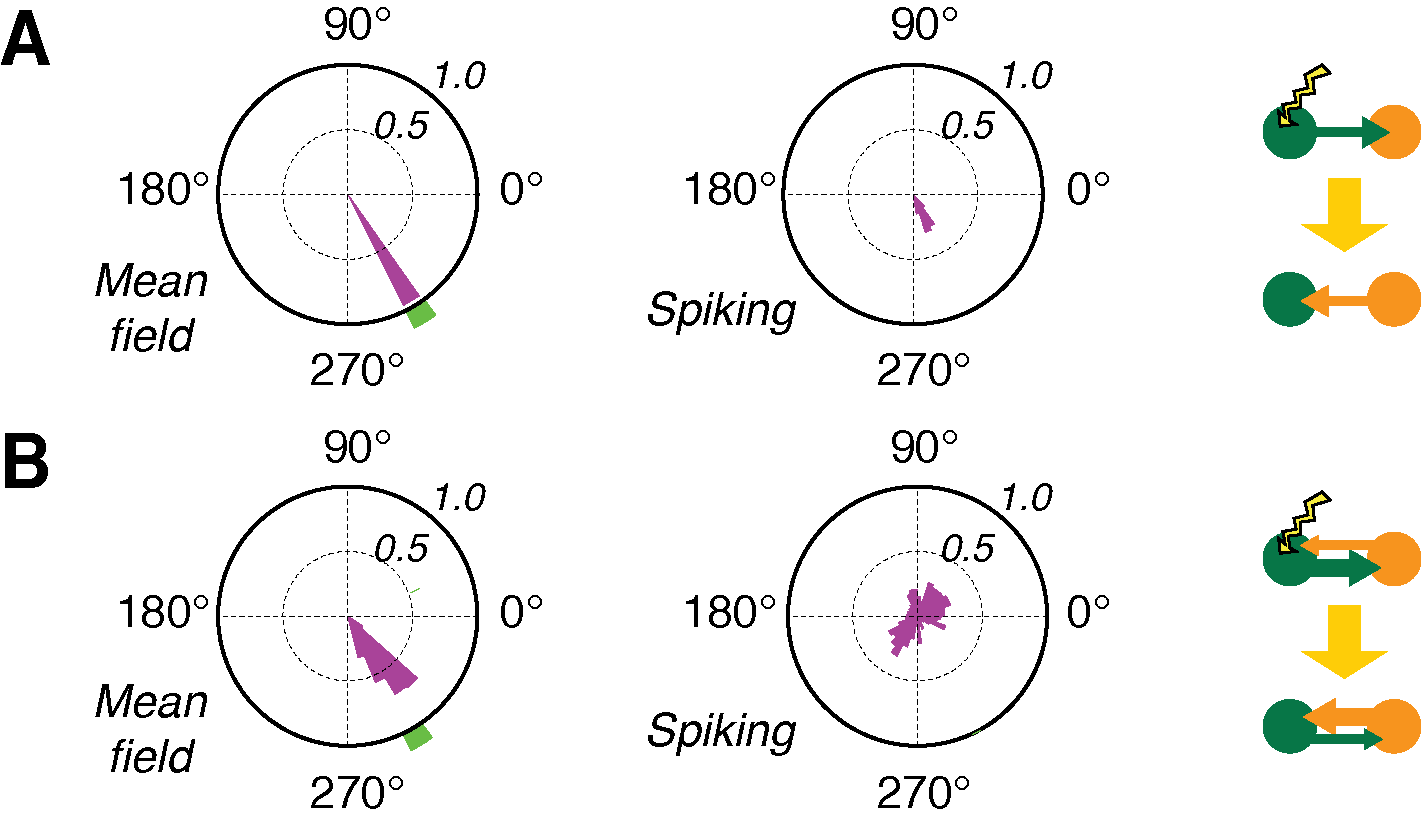

Supplement: Figure S2 — Dynamic control of effective connectivity (perturbation applied to the leader area). A–B: frequency histogram of successful switching for pulses applied at different phases ( for the rate model and for the network model). Predicted intervals for successful switching are marked in green, for the unidirectional (panel E) and for the leaky effective driving (panel F) motifs (left, rate model; right, network model; parameters as in Figures 3 and 4). Diagrams of the induced transitions are shown in the third column (see Figure 6 for perturbations applied to the laggard area). (TIFF) [file pcbi.1002438.s002.tiff]

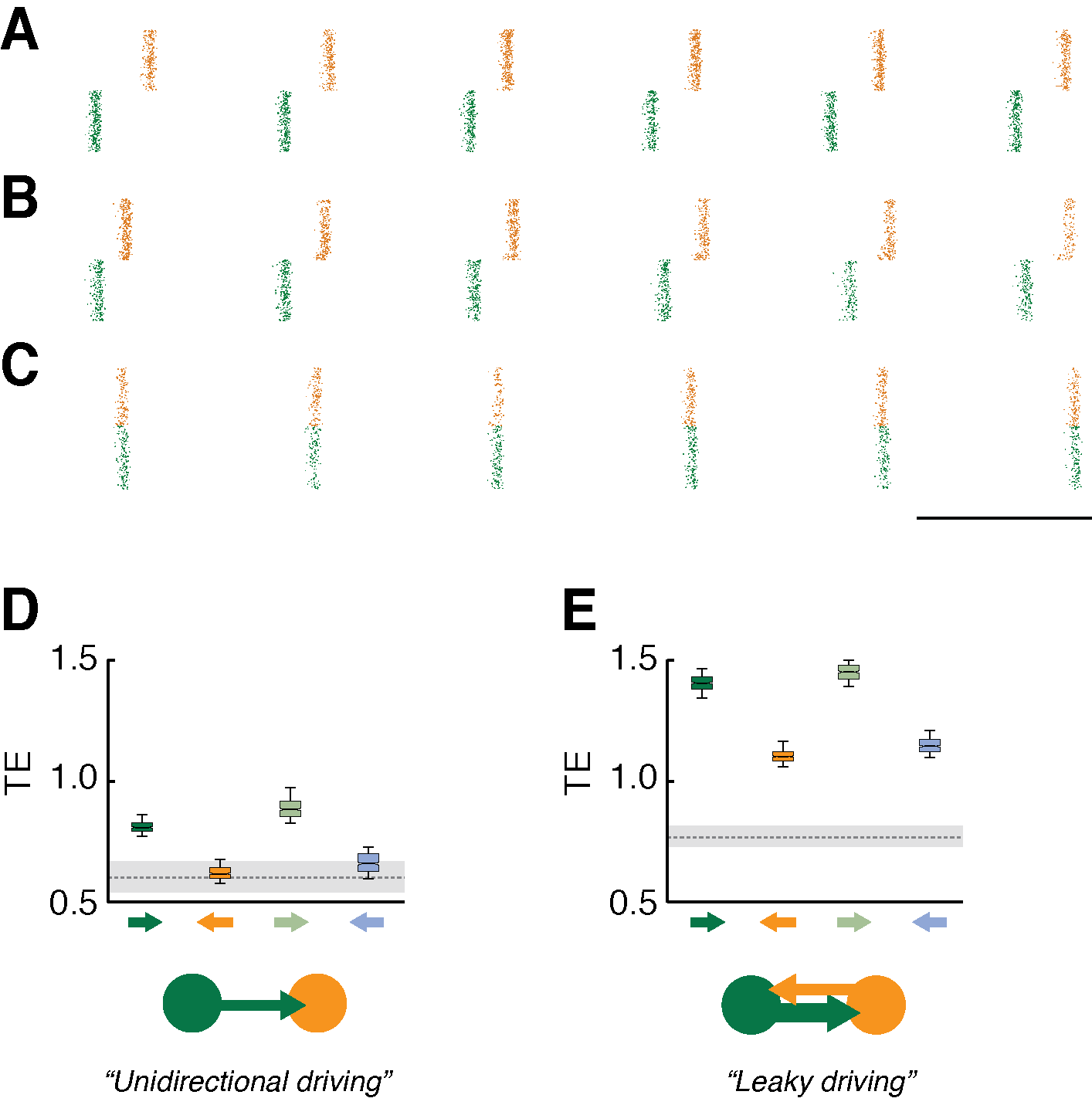

Supplement: Figure S3 — Effective connectivity with transmission lines (TLs). We consider a fully symmetric structural motif of structurally connected areas with embedded unidirectional TLs. Synapses involved in TLs are enhanced by multiplying the ordinary excitatory peak conductance by a multiplier . Raster plots relative to the spiking activity of excitatory neurons of the two areas are shown in panels A–C (green and orange color denote spikes of excitatory neurons from different populations, the horizontal scale line corresponds to ) for a weak inter-areal coupling (unidirectional driving effective motif, see Figure 3 for parameters). A: when (no TL embedded), the synchronous oscillations of the two populations lock in an out-of-phase fashion. B: for (just below a critical value), the raster plot of the spiking activity is virtually indistinguishable from the raster plot of panel A. C: for (just above a critical value), the oscillations of the two populations lock in an in-phase configuration. D–E: Effective connectivities associated to different dynamical states are measured by Transfer Entropy (TE), evaluated from “LFPs” time-series, for all possible directed interactions (indicated by green or orange arrows). Boxes indicate the interquartile range and whiskers the confidence interval for the estimated TEs. TEs above the grey horizontal band indicate statistically significant causal influences (see Methods ). In each plot, the third and the fourth boxes (from left to right) refer to TEs evaluated from “LFPs” restricted to groups of neurons that are source and target of a TL (pale green color denotes TL in the “green-to-orange” area direction, lilac color denotes TL in the “orange-to-green” area direction). Below each TE box-plot, effective connectivity is also represented in a diagrammatic form. Arrow thicknesses encode the strength of corresponding causal interactions (if statistically significant). D: TEs for the unidirectional driving effective motif with embedded TLs (). E: TEs fo [file pcbi.1002438.s003.tiff]

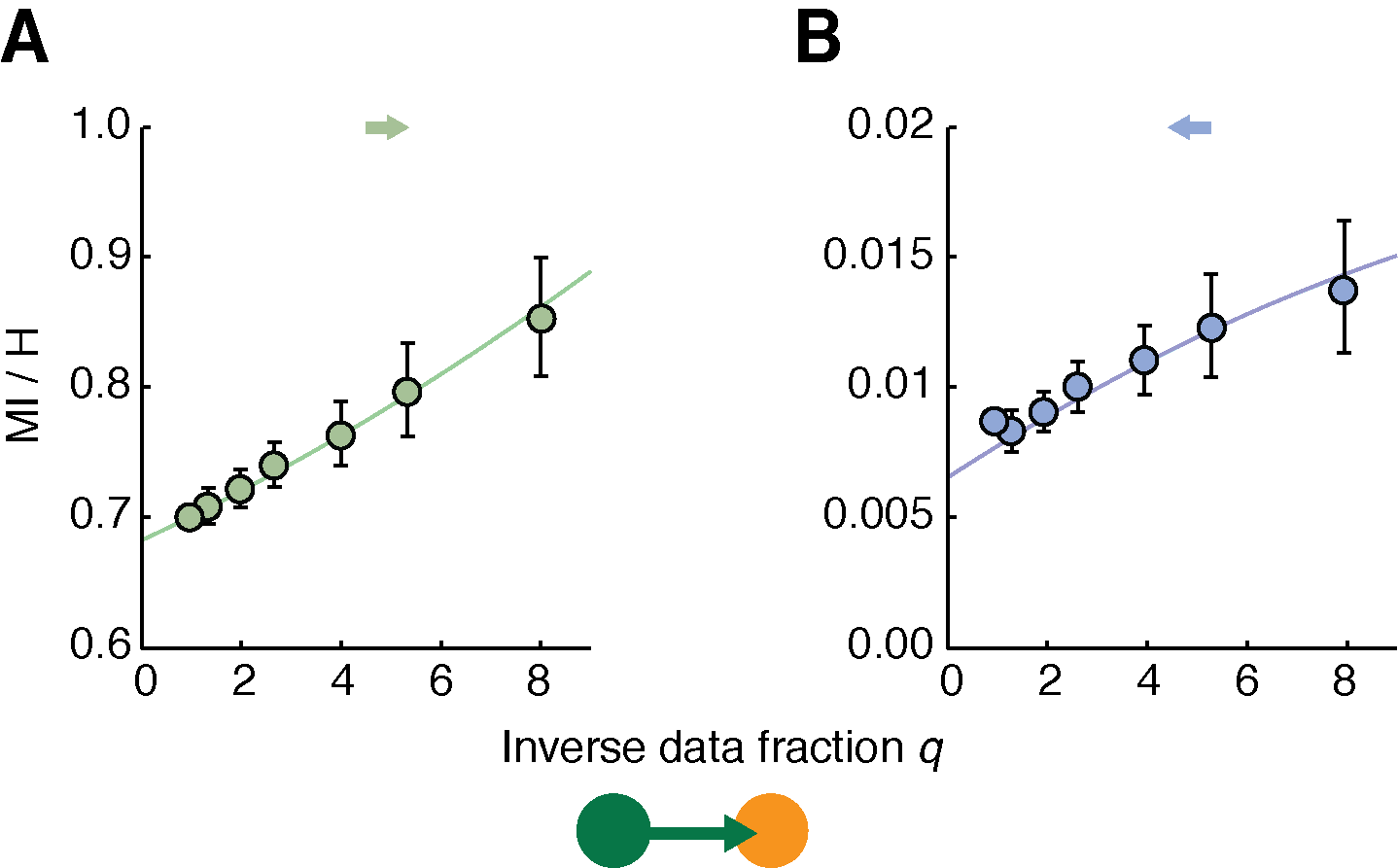

Supplement: Figure S4 — Scaling of Mutual Information (MI) with spike train length. MI normalized by entropy (at optimal time lag) is plotted against the inverse data fraction . For each data fraction , several bivariate spike trains are extracted from the original long spike trains (, ) and the mean MI is further averaged over these reduced-length spike trains. Asymptotic values are extrapolated through a quadratic interpolation. Error bars correspond to standard error. A: unidirectional driving effective motif, MI along the TL in the leader-to-laggard direction (pale green color), extrapolated asymptotic value is . B: unidirectional driving effective motif, MI along the TL in the laggard-to-leader direction (lilac color), extrapolated asymptotic value is . In both cases, the finite size of the used spike trains produces a positive but small bias in the estimation of MI. Compared to Figure 8C, for the leader-to-laggard direction the overestimation is of and for the laggard-to-leader direction is of . (TIFF) [file pcbi.1002438.s004.tiff]
