# Supplementary material for: Dynamic Effective Connectivity of Inter-Areal Brain Circuits
Source: PLoS Comput Biol. 2012 Mar 22;8(3):e1002438. doi: 10.1371/journal.pcbi.1002438 (PMC3310731; doi:10.1371/journal.pcbi.1002438)
Supplement: Text S1 — Full description of model parameters and complete analytic expressions. This text contains the following sections: i) Model neurons; ii) Model synapses; iii) Parameters of the background noise; iv) Phase response of the rate model; v) Phase-locking in the rate model. (PDF) [file pcbi.1002438.s005.pdf]

Supporting Text S1

Model neurons

We use the Wang-Buzski (WB) conductance-based model (ref. **[86]** in main text) to describe each single excitatory and inhibitory neuron. The WB model is described by a single compartment endowed with sodium and potassium currents. The membrane potential is given by:

$$C\frac{dV}{dt} = -I_L - I_{Na} - I_K + I_{ext} + I_{rec}$$

where C is the capacitance of the neuron, $I_L = g_L(V - V_L)$ is the leakage current, I_{ext} is an external driving current and I_{rec} is due to recurrent interactions with other neurons in the network (see later). Sodium and potassium currents are voltage-dependent and given by $I_{Na} = g_{Na}m_\infty^3 h(V - V_{Na})$ and $I_K = g_K n^4(V - V_K)$. The activation of the sodium current is instantaneous:

$$m_\infty(V) = \frac{\alpha_m(V)}{\alpha_m(V) + \beta_m(V)}$$

Sodium current inactivation and potassium current activation evolve according to:

$$\frac{dx}{dt} = \Phi \cdot (\alpha_x(V)(1 - x) - \beta_x(V)x)$$

where $x = h, n$ and α_x and $\beta_x(V)$ are non-linear functions of the membrane potential given by:

$$\begin{aligned}
\alpha_m(V) &= \frac{0.1(V + 35)}{1 + e^{-\frac{V+35}{10}}} \\
\beta_m(V) &= 4e^{-\frac{V+60}{18}} \\
\alpha_n(V) &= \frac{0.03(V + 34)}{1 - e^{-\frac{V+34}{10}}} \\
\beta_n(V) &= 0.375e^{-\frac{V+44}{80}} \\
\alpha_h(V) &= 0.21e^{-\frac{V+58}{20}} \\
\beta_h(V) &= \frac{3}{1 + e^{-\frac{V+28}{10}}}
\end{aligned}$$

Other parameters are $g_{Na} = 35$ mS/cm^2, $V_{Na} = 55$ mV, $g_K = 9$ ms/cm^2, $V_K = -90$ mV, $g_L = 0.1$ mS/cm^2, $C = 1$ μF/cm^2 and $\phi = 5$.

Model synapses

The synaptic current induced in a postsynaptic neuron by a single presynaptic action potential is given by $I_{spike}(t) = -g_x s_{spike}(t)(V - V_x)$, where V is the potential in the postsynaptic neuron and V_x is the reversal potential of the synapse (for excitatory synapses $V_E = 0$ mV, for inhibitory synapses $V_I = -80$ mV). The time-course of the postsynaptic conductance is described by:

$$s_{spike}(t) \propto (\exp(-(t + d - t^*)/\tau_1) - \exp(-(t + d - t^*)/\tau_2))$$

for $t > t^*$, 0 otherwise, where t^* is the time of the presynaptic spike, d is the latency, τ_1 the rise-time and τ_2 the decay-time. The total recurrent current $I_{rec}(t)$ is the sum of time-dependent contributions $I_{spike}(t)$ from all the presynaptic spikes fired to time t. The normalization constant of $s_{spike}(t)$ is chosen such as the peak value of s_{spike} is equal to 1. For all simulations in the paper, we take $\tau_1 = 1$ ms, $\tau_2 = 3$ ms and $d = 0.5$ ms. Thus, post-synaptic currents have a relatively fast decay, corresponding to AMPA-like excitatory and GABA$_A$-like inhibitory synapses. For simplicity, we take only two possible peak conductances, $g_I = 90$ μS/cm^2 for inhibitory synapses within an area and $g_E = 5$ μS/cm^2 for excitatory synapses within and between areas.

Parameters of the background noise

In addition to recurrent synaptic inputs, each neuron receives a noisy input, representing background spiking activity. It is modeled as an excitatory current having the same functional form of a recurrent current induced by a Poisson spike train with firing rate f_{ext}. The peak conductance of this noisy background input is g_{ext}. In our simulations, we take $f_{ext} = 5$ kHz, and $g_{ext} = g_E = 5$ μS/cm^2. Each neuron is driven by statistically independent Poisson noise realizations.

Phase response of the rate model

As previously throughly reported in the Supplementary Material of ref. **[42]** (in main text), the firing rate of a single oscillating area (only local inhibitory coupling $K_I < 0$ with delay D) can be derived analytically assuming that: (i) the total input current $I_{tot}(t) = I + K_I R(t - D)$ is below threshold (i.e. negative) for a duration $T_{st} > D$; (ii) the delay D and the oscillation period T fulfill the inequalities $D < T - T_{st} < 2D$. The conditions (i) and (ii) hold for sufficiently strong local inhibition, and, specifically, for the value $K_I = -250$ and the delay $D = 0.1$ adopted in the main paper. Under these conditions, the limit cycle of the firing rate assumes then the following analytic form (see Figure 2B in the main paper):

$$R(t) = R_{peak} \cdot \begin{cases} e^{-t} & t \in [0, T_{st}] \\ e^{-t} + K_I e^{D} \left[e^{-t} - e^{-T_{st}} + e^{-t}(t - T_{st}) \right] & t \in [T_{st}, T_{st} + D] \\ e^{-t} + K_I e^{D} \left[e^{-t} - e^{-T_{st}} + e^{-t}(t - T_{st}) \right] & \\ \quad + K_I^2 e^{2D} \left[e^{-t} - e^{-D-T_{st}} + e^{-t}(t - T_{st} - D + \frac{(t-T_{st}-D)^2}{2}) \right] & t \in [T_{st} + D, T] \end{cases}$$

where R_{peak} is the peak amplitude of the periodic oscillation of the rate and depends linearly on the level of the background current I. The oscillation period T and the sub-threshold time can be determined numerically by solving the system of non-linear equations:

$$\begin{aligned} e^{T-T_{st}} &= 1 + K_I e^{D} \left(1 + T - T_{st} - D - e^{T-T_{st}-D} \right) \\ e^{T} &= 1 + K_I e^{D}(1 - e^{T-T_{st}} + T - T_{st}) + K_I^2 e^{2D} \left(1 - e^{T-T_{st}-D} + T - T_{st} - D + \frac{(T - T_{st} - D)^2}{2} \right) \end{aligned}$$

We define the phase relative to the oscillation as $\phi(t) = \mod(t - t_0, T)$, where the time-shift t_0 is chosen such as $\phi(t_{t_peak}) = 0$ in correspondence of the timings t_{peak} of oscillation peaks. Phases are therefore, with this notation, bounded between 0 and 1. We use this convention throughout all analytic developments for the sake of simplicity. In The results involving phases in the main article are then translated back into the more usual angular range comprised between 0° and 360°. The application of a pulse current $\delta I = h\delta(\phi - \phi_p)$ at a phase ϕ_p induces a phase-shift $\delta\phi(\phi_p) = hZ(\phi_p)$ (see Figure S3B). The analytic expression for the Phase Response Curve (PRC) $Z(\phi)$ can be derived from the knowledge of the limit cycle solution, and reads:

$$Z(\phi) = R_{peak} \cdot \begin{cases} 0 & \phi \in [0, \phi_{st}] \\ -e^{T(\phi-1)} \left(1 + K_I T e^{D}(1 - \phi - \phi_D) \right) & \phi \in [\phi_{st}, 1 - \phi_D] \\ -e^{T(\phi-1)} & \phi \in [1 - \phi_D, 1] \end{cases}$$

where $\phi_{st} = \frac{T_{st}}{T}$ and $\phi_D = \frac{D}{T}$. The resulting PRC is therefore null over a very large interval of phases, leading in this broad range to refractoriness toward perturbations. A plot of $Z(\phi)$ for the parameters used in our study is reported in Figure 4D (main text).

Phase-locking in the rate model

As discussed in the main text, the time-evolution of the instantaneous phase shift $\Delta\phi(t)$ between two coupled areas can be described, in the weak coupling limit, by the equation:

$$\frac{d\Delta\phi}{dt} = \Gamma(\Delta\phi)$$

The term $\Gamma(\Delta\phi)$ is a functional of the phase response and of the limit cycle waveform of the uncoupled oscillating areas. In terms of the previously derived analytic expressions of $Z(\phi)$ and of the rate oscillation limit cycle $R(\phi)$ (phase-reduced) for $K_E = 0$, this functional can be expressed as $\Gamma(\Delta\phi) = C(\Delta\phi) - C(-\Delta\phi)$, where:

$$C(\Delta\phi) = \int_0^1 Z(\phi) R(\phi + \Delta\phi - D) d\phi$$

Stable phase-lockings are therefore given by the zeroes of Γ with negative slope crossing. Analytic expressions for the integral $C(\Delta\phi)$ have already been derived and published in the Supplementary Material of ref. **[31]** (in main text). We report here these expression again, in order to make the presentation of results self-contained. To compute $C(\Delta\phi)$, six different intervals of $\Delta\phi$ need to be considered separately. The result is:

$$C(\Delta\phi) = \begin{cases} C_{00}(\phi_{st},1) + C_{10}(\phi_{st},1-\phi_D) & \Delta\phi \in [\phi_D - \phi_{st}, \phi_{st} + \phi_D - 1] \\ \\ C_{00}(\phi_{st},1) + C_{10}(\phi_{st},1-\phi_D) + C_{01}(\phi_{st}+\phi_D-\Delta\phi,1) & \Delta\phi \in [\phi_{st}+\phi_D-1, \phi_{st}+2\phi_D-1] \\ \\ C_{00}(\phi_{st},1) + C_{10}(\phi_{st},1-\phi_D) + C_{01}(\phi_{st}+\phi_D-\Delta\phi,1) & \\ \quad +C_{11}(\phi_{st}+\phi_D-\Delta\phi,1-\phi_D) + C_{02}(\phi_{st}+2\phi_D-\Delta\phi,1) & \Delta\phi \in [\phi_{st}+2\phi_D-1, \phi_D] \\ \\ C_{00}(\phi_{st},1+\phi_D-\Delta\phi) + e^T C_{00}(\phi_{st}+\phi_D-\Delta\phi,1) & \\ \quad +C_{10}(\phi_{st},1-\phi_D) + C_{01}(\phi_{st},1+\phi_D-\Delta\phi) & \\ \quad +C_{11}(\phi_{st},1-\phi_D) + C_{02}(\phi_{st}+2\phi_D-\Delta\phi,1+\phi_D-\Delta\phi) & \Delta\phi \in [\phi_D, \phi_{st}-1+3\phi_D] \\ \\ C_{00}(\phi_{st},1+\phi_D-\Delta\phi) + e^T C_{00}(\phi_{st}+\phi_D-\Delta\phi,1) & \\ \quad +C_{10}(\phi_{st},1-\phi_D) + C_{01}(\phi_{st},1+\phi_D-\Delta\phi) & \\ \quad +C_{11}(\phi_{st},1-\phi_D) + C_{02}(\phi_{st}+2\phi_D-\Delta\phi,1+\phi_D-\Delta\phi) & \\ \quad +C_{12}(\phi_{st}+2\phi_D-\Delta\phi,1-\phi_D) & \Delta\phi \in [\phi_{st}-1+3\phi_D, 2\phi_D] \\ \\ C_{00}(\phi_{st},1+\phi_D-\Delta\phi) + e^T C_{00}(\phi_{st}+\phi_D-\Delta\phi,1) & \\ \quad +C_{10}(\phi_{st},1+\phi_D-\Delta\phi) + e^T C_{10}(1+\phi_D-\Delta\phi,1-\phi_D) & \\ \quad +C_{01}(\phi_{st},1+\phi_D-\Delta\phi) + C_{11}(\phi_{st},1+\phi_D-\Delta\phi) & \\ \quad +C_{02}(\phi_{st},1+\phi_D-\Delta\phi) + C_{12}(\phi_{st},1+\phi_D-\Delta\phi) & \Delta\phi > 2\phi_D \end{cases}$$

where

$$\begin{aligned}
C_{00}(a,b) &= -(b-a)Te^{-T(1-\Delta\phi+\phi_D)} \\
C_{10}(a,b) &= K_I e^{T(2\phi_D-1-\Delta\phi)}\left[\frac{T(x+\phi_D-1)^2}{2}\right]_a^b \\
C_{01}(a,b) &= -K_I e^{D-T}\left[T(b-a)e^{T(\phi_D-\Delta\phi)} - e^{-T_{st}}(e^{bT}-e^{aT}) + e^{T(\phi_D-\Delta\phi)}\left[\frac{T(x+\Delta\phi-\phi_D-\phi_{st})^2}{2}\right]_a^b\right] \\
C_{02}(a,b) &= -K_I^2 e^{2D-T}\Big[(b-a)e^{T(\phi_D-\Delta\phi)} - e^{-D-T_1}(e^{bT}-e^{aT}) + \\
&\quad + e^{T(\phi_D-\Delta\phi)}\left[\frac{T(x+\Delta\phi-2\phi_D-\phi_{st})^2}{2} + \frac{T(x+\Delta\phi-2\phi_D-\phi_{st})^3}{6}\right]_a^b\Bigg] \\
C_{11}(a,b) &= K_I^2 e^{2D-T}\left[e^{T(\phi_D-\Delta\phi)}\left(\frac{(bT)^2}{2} - \frac{(aT)^2}{2} + T(D-T)(b-a)\right) - \right. \qquad (1) \\
&\quad -e^{-T_{st}}\left[(xT-1)e^{xT} + (D-T)e^{xT}\right]_a^b + \\
&\quad \left. + e^{T(\phi_D-\Delta\phi)}\left[\frac{(xT+D-T)^3}{3} + T(x+\Delta\phi-2\phi_D-\phi_{st})\frac{(xT+D-T)^2}{2}\right]_a^b\right] \\
C_{12}(a,b) &= K_I^3 e^{3D-T}\left[e^{T(\phi_D-\Delta\phi)}\left(\frac{(bT)^2}{2} - \frac{(aT)^2}{2} + T(D-T)(b-a)\right) - \right. \qquad (2) \\
&\quad -e^{-D-T_{st}}\left[(xT-1)e^{xT} + (D-T)e^{xT}\right]_a^b + \\
&\quad + e^{T(\phi_D-\Delta\phi)}\left[\frac{(xT+D-T)^3}{3} + T(1+\Delta\phi-3\phi_D-\phi_{st})\frac{(xT+D-T)^2}{2} + \frac{(xT+D-T)^4}{8} + \right. \\
&\quad \left.\left. + T(1+\Delta\phi-3\phi_D-\phi_{st})\frac{(xT+D-T)^3}{3} + T(1+\Delta\phi-3\phi_D-\phi_{st})^2\frac{(xT+D-T)^2}{4}\right]_a^b\right]
\end{aligned}$$

where $[f(x)]_a^b = f(b) - f(a)$. A plot of $\Gamma(\Delta\phi)$ for the parameters used in our study is reported in Figure 4B (main text).
